# Knowledge of Lassa fever, its prevention and control practices and their predictors among healthcare workers during an outbreak in Northern Nigeria: A multi-centre cross-sectional assessment

**DOI:** 10.1371/journal.pntd.0010259

**Published:** 2022-03-14

**Authors:** Yusuf Hassan Wada, Ibrahim Abayomi Ogunyinka, Kazeem Babatunde Yusuff, Chinwe Lucia Ochu, Mohammed Yahaya, Garba Mohammed Khalid, Yahkub Babatunde Mutalub, Sulaiman Badmus Adeniye

**Affiliations:** 1 Society for Family Health, Abuja, Nigeria; 2 Department of Clinical Pharmacy and Pharmacy Practice, Faculty of Pharmaceutical Sciences, Usmanu Danfodiyo University, Sokoto, Nigeria; 3 Department of Clinical Pharmacy and Practice, College of Pharmacy, QU Health, Qatar University, Doha, Qatar; 4 Prevention, Programmes and Knowledge Management, Nigeria Centre for Disease Control, Abuja, Nigeria; 5 Department of Medical Microbiology and Parasitology, Usmanu Danfodiyo University, Sokoto, Nigeria; 6 Department of Pharmaceutics and Pharmaceutical Technology, Bayero University, Kano, Nigeria; 7 Department of Clinical Pharmacology, College of Medical Sciences, Abubakar Tafawa Balewa University, Bauchi, Nigeria; 8 Maributh Global Resources Limited, Sagamu, Nigeria; University of Heidelberg, GERMANY

## Abstract

**Background:**

The year 2020 Lassa fever (LF) outbreak had the greatest disease burden and this can place an enormous strain on the already overstretched healthcare system and can potentially increase morbidity and mortality due to infectious diseases.

Therefore, having a knowledgeable healthcare workforce with appropriate skills and competencies to prevent and manage outbreaks of a neglected infectious disease such as LF in Nigeria will potentially enhance public health. Thus, this survey assessed the level of knowledge of LF and its prevention and control (PC) measures amongst the healthcare workers (HCWs) during a LF outbreak in Katsina state, Nigeria.

**Methodology/Principal findings:**

During this cross-sectional survey, HCWs complete a validated 29-item questionnaire comprising 18 items on the knowledge of LF and its PC measures and an item on global self-evaluation of their LF knowledge. Psychometric properties of the questionnaire were evaluated. Chi-square and binary logistic regression analyses were conducted.

Out of 435 HCWs invited, a total of 400 participated in the study (92% response rate). The majority of participants (51.8%) demonstrated inadequate LF knowledge, with 62.9% of those scoring low having a high self-perception of their LF knowledge with the global scale. This LF knowledge over-estimation was predicted by LF training status (odds ratio (OR) 2.53; 95% CI: 1.49–4.30; *p* = 0.001). The level of LF knowledge and its PC measures among the study participants was low (11.60±8.14, 64.4%) and predicted by participants’ LF training status (OR 2.06; 95% CI: 1.19–3.57; *p* = 0.009), place of work (OR 1.82; 95% CI: 1.07–3.08; *p* = 0.03) and their designations (OR 2.40; 95% CI: 1.10–5.22; *p* = 0.03).

**Conclusion:**

The level of knowledge of LF and its PC measures among the HCWs surveyed was suboptimal and participants’ LF training status, place of work and occupational category were the significant predictors. In addition, LF knowledge overestimation on a global scale was observed among a majority of HCWs and this was also predicted by LF training status. Therefore, there is a critical need for health authorities in Nigeria to prioritize continuous on-the-job training of HCWs on priority neglected tropical diseases such as Lassa fever.

## Author summary

In about five decades in Nigeria, Lassa fever (LF) has infected 3897 and caused 1319 deaths (including 71 healthcare workers (HCWs)) giving a case fatality rate of 33.8% for confirmed cases in Nigeria. Poor knowledge of LF and its prevention and control (PC) measures amongst HCWs can lead to non-adherence to LF safety protocols, healthcare-associated infections, poor patient outcomes, and even the death of infected HCWs. In addition, LF knowledge over-estimation may lead to mismanagement of LF cases with potential fatal consequences. Therefore, this study assessed if the nine different cadres of HCWs interviewed from four health facilities during an LF outbreak in Katsina, Nigeria have the appropriate level of LF knowledge. Using a validated questionnaire, data were collected from 400 HCWs and then analyzed with Statistical Package for the Social Sciences (SPSS) Version 25. Results were tabulated and associations between the variables determined. More than half of the HCWs demonstrated inadequate LF knowledge with nearly two out of three of these HCWs over-estimating their LF knowledge. However, adequate LF knowledge was predicted by training on LF and this in turn predicted LF knowledge over-estimation.

## Introduction

Lassa fever (LF) is caused by a biosafety level-4 virus known as Lassa virus (LASV), and it is a heterogeneous neglected zoonotic disease of the tropics [1,2]; that was first identified in the town of Lassa, Borno state, Nigeria in 1969 [3]. LF continues to impose significant clinical, economic and health security burdens on affected communities and countries. LF is endemic to Nigeria and some other West African countries, including the Mano River Union Countries of Sierra Leone, Liberia and Guinea [4], with sporadic imported cases been reported in Europe and North America [5]. However, Nigeria bears the highest burden of the disease in sub-Saharan Africa, and records of community and healthcare associated transmissions of LF between 1969 and 2021 in the country identified 25191 suspected cases, 3897 confirmed cases and 1319 deaths (including the death of 71 HCWs) [6–8]. The disease burden of LF surpasses those of other viral hemorrhagic fevers except for dengue and yellow fever [9]. In addition, LF is the most frequently imported viral hemorrhagic disease to non-endemic settings [1].

The majority of the cases of LF are asymptomatic and mild (sub-clinical or self-limiting) but the severe disease is associated with a broad tissue tropism characterized by facial edema, bleeding, hypotension, acute kidney injury, severe anemia, confusion, coma [10–14], with death sometimes occurring within 14–20 days of symptom onset [9,15,16]. The primary mode of disease transmission is through human contact with contaminated urine or feces of infected rodents, particularly *Mastomys natalensis* (a natal multimammate mouse) [10,17]. Secondary LF transmission is via exposure of humans to body fluids of infected persons mostly in hospital settings and this has grave consequences for healthcare workers (HCWs) with inadequate knowledge of infection prevention and control practices [16,18–22]. Nigeria remains a home to many epidemic-prone diseases with Katsina state simultaneously battling with COVID-19, cerebrospinal meningitis, yellow fever and measles [23]. The year 2020 LF outbreak in Nigeria recorded the highest disease burden and death toll in five decades, including affecting the highest number of HCWs (48) [7]. It was also the second time Katsina state was reporting an LF outbreak, where two died and one HCW was affected [7]. During the first LF outbreak in Katsina state, Chinaka (2016) reported that only eight per cent of the HCWs surveyed had good knowledge of LF [24]. In addition, data on LF knowledge and its prevention and control (PC) measures among HCWs in Northern Nigeria are scarce, despite the endemicity of the disease in the region [10].

LF outbreaks have been significantly linked to hospital-acquired transmissions and poor patient outcomes [18,6,25,26], with HCWs’ poor knowledge of LF and its PC measures identified as a major underlining factor [27–29]. Consequently, an assessment of knowledge of LF and its PC measures among HCWs is crucial in ensuring that optimal patient outcomes are not compromised during the management of outbreaks, and in designing appropriate interventions to control the transmission and treatment of LF [25,30–32]. Further, HCWs are at the highest risk of contracting and transmitting LF because of their frequent contact with infected patients and blood or body fluids [18,26]. In addition, HCWs are active behavioral change agents whose contributions are crucial in promoting compliance with disease preventive measures among the populace using evidence-based risk-communication and community engagement strategies [30]. Therefore, the objective of this study was to assess the knowledge of LF, its prevention and control (PC) measures and their predictors among HCWs during an outbreak in Katsina state.

## Material and methods

### Ethics statement

The study was approved by the Health Research Ethical Review Committee, Katsina State Ministry of Health (MOH/ADM/SUB/1152/1/341). Permission to conduct the study was also obtained from the office of the Chief, Medical Advisory Committee of the selected tertiary health facility and Medical Directors of the respective secondary health facilities. Written informed consent of the study participants was obtained by requesting each participant to tick (√) a Yes-box in the introductory page of the anonymized questionnaire. This was done after the objectives and benefits of the study had been fully explained to them. Participants were also informed that non-participation will not attract any penalty and that they were free to withdraw at any time during the course of the interview. They were also assured that the information provided will be used solely for research purposes. The authors assert that all procedures contributing to this work comply with the Declarations of Helsinki and the ethical standards of the relevant national guidelines on human experimentation.

### Study design and setting

This was a cross-sectional study involving 400 healthcare workers sampled from four public health facilities in Katsina state, Nigeria: Federal Medical Centre, General Hospital, Turai Umaru Musa Yar’adua Maternity Hospital and General Ahmadi Rimi Specialist Hospital; between April 15, 2020 and May 28, 2020. The former is a tertiary hospital and the others are secondary. Katsina state is located in the Sahel Savanna in the North-western zone of Nigeria with a population of over 5.8 million residents spread over 3 senatorial districts and 34 local government areas [33]. Katsina state is the fourth most populous state in Nigeria and is bordered by Kano (to the East) and Kaduna (to the South). All the states experienced an outbreak of Lassa fever in 2020 [7].

### Study population

Healthcare workers refer to those whose primary intent of their actions is to enhance health [34]. Therefore, the study participants included medical doctors, dentists, pharmacists, nurses, midwives, radiographers, medical laboratory scientists, community health extension workers (CHEWs) and hospital aides who were staff members at the selected study sites.

### Questionnaire development and validation

An initial 63-item questionnaire was developed after extensive search of published literature in the research area [29,35,36,37], and it comprised of three sections-demographic characteristics, knowledge of LF, its control and prevention practices.

The psychometric properties of the questionnaire were evaluated with face and content validity, and reliability analysis using Cronbach’s alpha and intra-class correlation coefficients (ICC). Factor analysis was also performed.

### Face validity

The face validity was assessed by five staff members of the Departments of Public Health, Ministries of Health, Katsina (2), Bauchi (2) and; NCDC (1) with relevant research experiences in the fields of public health and infectious disease. They were requested to comment on the difficulty level, relevance and ambiguity of the 63 items of the questionnaire (qualitatively) and then rank the importance /significance of each item using a five-point Likert scale (1 meaning “absolutely unimportant” to 5 meaning “absolutely important”) and items having impact factor score below 1.5 were removed from the questionnaire [38].

### Content validity

Content validity of the resulting 48-item questionnaire was assessed by six experts (clinical pharmacist, pharmacologist and microbiologist; epidemiologist; environmental health expert and infectious disease physician) with a 4-point Likert scale (1-not relevant to 4-very relevant) for relevancy, and 3-point Likert scale each (1-not clear to 3-very clear) for clarity and essentiality (1-not essential to 3-essential). The experts assessed whether each item was clear (understandable), relevant (important) and essential (necessary) and these criteria were met for all the items retained in the questionnaire. Quantitatively, the relevancy of each of the 48 items and the scale (considering the 48 items as a group) was assessed using Item-content validity index (Item-CVI) and Scale-content validity index (Scale-CVI) respectively. Items with Item-CVI values above 0.79 were retained (considered relevant) [39]. Scale-CVI was determined using the Average CVI method and values of 0.90 and above were considered to have excellent content validity [39]. Kappa statistic was also calculated to check if the Item-CVI values obtained were robust to errors due to inflated values or chance agreement among the experts [40], and values above 0.74 were considered excellent (the degree of expert agreement is beyond chance). The essentiality of the items was assessed using content validity ratio and items with values less than 0.99 removed [41]. The clarity of each item and the scale was also determined, and values from 2.33 to 3.00 for both individual items and the scale were considered clear [42].

Qualitatively, the experts suggested some modifications including rephrasing and reordering (change of item sequence). These modifications resulted in retaining 29 items that were considered clear, relevant and essential. Thus, the final 29-item questionnaire comprised 7 items on demographic characteristics and 22 items related to awareness and knowledge.

### Reliability

The reliability of the 18-item LF knowledge scale was evaluated using internal consistency and stability methods. The internal consistency was assessed using Cronbach’s alpha coefficient (α) with a value of 0.7 considered adequate [43]; while for the temporal stability, an intraclass correlation coefficient (ICC) of 0.7 was also considered adequate [44].

### Factor analysis

Factor analysis was performed in order to determine the underlying factor-structure of the 18-item knowledge section of the questionnaire. The data collected was first assessed for its suitability for factor analysis by inspecting the survey’s sample size (it should not be less than 300 participants), correlation matrix (substantial number of coefficients >0.3 should be found), Bartlett’s test of sphericity (should be significant: *p* <0.05) and Kaiser-Meyer-Olkin index (should have a value of ≥ 0.6) [45]. Second, factor extraction was performed using Principal Components Analysis (PCA) which involved the use of the following techniques: Kaiser’s criterion (retaining components with eigenvalues >1), scree test (retaining components above the break or “elbow” of the plot) and parallel analysis (retaining components with eigenvalues greater than that produced by parallel analysis). After the number of factors to be retained was determined, Oblimin Rotation was then applied to aid its interpretation. The final version of the 29-item questionnaire had two sections: A- demographics comprising 7 items and B- 18 items related to knowledge of LF, 2 to awareness, 1 to LF training status and 1 on self-rating of LF knowledge (see Supporting Information for a copy of the questionnaire: S1 Questionnaire).

### Sample size determination

Using an online sample calculator, G*Power version 3.1.9.7 [46]; the sample size was determined using power analysis. By pre-specifying the significance level (α) as 0.05, effect size (d) as 0.25, power level as 0.95 and number of groups as 4; a sample size of 280 was obtained and adding 15% contingency for non-response made it 322 participants. However, we included 400 participants for the study.

### Eligibility criteria

Only those with tenure appointments, consented and willing to complete the questionnaire before the close of work on the appointed dates of questionnaire administration were recruited for the study.

### Sampling techniques and data collection procedure

The study sites were chosen purposively. Between them, the four study sites have the largest number of HCWs, enjoy the most client/patient patronage, covers the senatorial district comprising the 2 Local Government Areas with the highest prevalence of LF in the state during the 2020 LF outbreak and all LF cases were managed in at least one of the selected study sites. A staff list was obtained from the respective hospital secretaries, and all the identified dentists, radiographers and medical laboratory scientists were interviewed due to their relatively small numbers, while stratified random sampling technique was used to recruit the other categories of HCWs.

Pilot-testing of the questionnaire was done with thirteen HCWs. The pilot-test data were excluded from the final analysis. Four hundred HCWs gave their consents and participated while thirty-five HCWs declined. Completed questionnaires were collected at the study site while follow up visits were made to HCWs who did not return the filled questionnaires at the first visit. Similarly, when a questionnaire was not completely filled (especially the knowledge section), follow up visits were made to ensure completion. This ensured a completion rate of 100%.

### Data management

Of the 18 items on knowledge, 12 items were assessed on a 3-point Likert scale (Yes, I agree; I am not sure and No, I disagree) while the remaining items were assessed with multiple choice questions.

Correct responses were scored as + 1 and 0 for incorrect responses. The response “I am not sure” and no response (blanks) were also scored as 0. The maximum possible score was 18 and those who scored 13 and above (> 71%) were considered to have adequate (good) knowledge and those who scored 12 and below inadequate (poor) knowledge [47]. Correct answers were based on information from National Guidelines for Lassa Fever Case Management [35] and WHO document [37]. Participants’ self-perceived LF knowledge was assessed with the following item: “How would you rate your knowledge of Lassa fever?” on a global scale ranging from 1 (Poor), through 2 (Average), 3 (Good), 4 (Very good) to 5 (Excellent) [48]. Those who perceived their LF knowledge to be from good to excellent were reclassified as having a “High” self-perception of their LF knowledge and those who self-rated themselves as being average or poor were reclassified as having a “Low” self-perception of their LF knowledge.

In addition, LF knowledge differentials were calculated for each participant that was trained on LF with the values obtained categorized as LF knowledge underestimation, accurate estimation and overestimation. The differentials were obtained by initially providing a common metric for both perceived (score obtained with the self-rated global scale) and actual LF knowledge (participant’s LF knowledge score from the 18 items), which was by converting actual knowledge to 5 since the maximum score for perceived LF knowledge is 5 [49]. After which, the actual knowledge scores were subtracted from the perceived knowledge scores for each participant. Due to the documentation of the adverse impacts of knowledge overestimation on patient outcome, the differential values were then reclassified (from under-, accurate and over-estimation) into overestimation and no overestimation. In this study, the actual LF knowledge was considered the *real* or *objective* LF knowledge of the participants.

### Statistical analysis

Data were analyzed using IBM SPSS statistics software, version 25.0 (IBM Corporation, Armonk, NY, USA: released 2017). The demographic profile, awareness of LF, main information source, and LF training status were the independent (predictor) variables while knowledge of Lassa fever and its PC measures and LF knowledge overestimation were the dependent (response) variable. The demographic profile was described with frequencies and proportions. Correct, incorrect and unsure/blank answers were calculated for each knowledge item. Chi-square test for independence was performed to determine the significant (*p*≤0.05) associations between the independent and dependent variables. Those that were found to be significant were then entered into a binary logistic regression model.

## Results

### Demographic profiles of participants

The response rate was 92% (400/435). The mean age (±standard deviation) and age range of participants was 34.00 (±8.54) years and 20–65 years respectively (following a non-significant Kolmogorov-Smirnov value). Most of the participants were males (70.5%; 282/400), nurses (20.3%; 81/400), practicing in tertiary care Centre (37.8%; 151/400) and within the last decade (72.8%; 291/400) (Table 1).

**Table 1:** Demographic profiles of the study participants.

Characteristics (N = 400)	Frequency (%)
**Age group (years)**	
2z0-29	144 (36.0)
30–39	160 (40.0)
40–49	71 (17.8)
≥ 50	25 (6.3)
**Sex**	
Male	282 (70.5)
Female	118 (29.5)
**Marital status**	
Single	102 (25.5)
Married	298 (74.5)
**Healthcare workers**	
Medical doctors	61 (15.3)
Dentists	20 (5.0)
Pharmacists	47 (11.8)
Laboratory scientists	30 (7.5)
Nurses	81 (20.3)
Midwives	56 (14.0)
Radiographers	14 (3.5)
CHEWs	40 (10.0)
^**1**^Attendants	51 (12.8)
**Place of work**	
GHK	120 (30.0)
FMC	151 (37.8)
TUMY Mat. Hosp.	86 (21.5)
GARSH	43 (10.8)
**Years of practice**	
1–10	291 (72.8)
11–20	76 (19.0)
≥21	33 (8.3)
**Educational status**	
Non-Graduates	204 (51.0)
Graduates	196 (49.0)

Mat., Maternity; Hosp., Hospital; CHEWs, Community Health Extension Workers; GHK, General Hospital, Katsina; FMC, Federal Medical Centre; TUMY, Turai Umar Musa Yar’adua; GARSH, General Ahmadi Rimi Specialist Hospital

^1^ Medical waste handlers-5, Physician aides-6, Dental health/surgery aides-10, Environmental health aides-8, Pharmacy aides-4, Ward aides-18

### Awareness, training on Lass fever and perceived knowledge of Lassa fever

Table 2 showed that only sixty-nine percent (276/400) of the participants were aware of an LF outbreak in their state of residence (Katsina) and their main source of information was colleagues, friends and family (38.0%; 105/276). Similarly, only sixty-nine percent (276/400) of the participants was ever trained on LF with majority self-rating their LF knowledge low on the global scale (50.4%; 139/276). In addition, each HCW occupational category have members that was trained on LF.

**Table 2:** Awareness, perceived knowledge and training on Lassa fever among healthcare workers.

Characteristics (N = 400)	Frequency (%)
**Awareness of LF outbreak**	
Yes	276 (69.0)
No	124 (31.0)
**Main information source (N = 276)**	
Formal training	104 (37.7)
Colleagues, family and friends	105 (38.0)
Media	67 (24.3)
**LF training status**	
Yes	276 (69.0)
No	124 (31.0)
**Perceived knowledge of LF**	
Excellent	68 (24.6)
Very good	41 (14.9)
Good	28 (10.1)
Average	66 (23.9)
Poor	73 (26.4)

### Psychometric properties of the questionnaire

The 18 items eliciting information on knowledge demonstrated good reliability (α = 0.734; 95% confidence interval-0.72–0.76) and stability (ICC = 0.73; 95% confidence interval-0.69–0.77).

The suitability of the collected data for *factor analysis* (Principal component analysis) was assured after inspection of the correlation matrix revealed the presence of many coefficients of 0.3 and above, the Kaiser-Meyer-Olkin index was 0.84 and Bartlett’s Test of Sphericity reached statistical significance (χ^2^ = 5541.20, *p* < 0.0005).

Principal components analysis revealed the presence of six components with eigenvalues exceeding 1, with all of them explaining a total of 67% of the variance. An inspection of the scree plot revealed a clear break after the third component. Using Catell’s scree test, three components (factors) were retained for further investigation. This decision was further supported by the results of Parallel Analysis, which showed only three components with eigenvalues exceeding the corresponding criterion values for a randomly generated data matrix of the same size (18 variables x 400 respondents).

The three-component (factor) solution explained a total of 48.5% of the variance. To aid in the interpretation of these three factors, Oblimin rotation was performed and this revealed symptoms-eliciting items loading substantially on component 1, transmission-eliciting on 2, and infection prevention and control-related item on 3 (Table 3). The correlation between the three factors was weak and ranged from r = -0.007, through 0.027 to 0.031 indicating that three different factors were measured.

**Table 3:** Factor loading results after Oblimin rotation.

Items	Factors		
	F1	F2	F3
**Factor 1 (F1): Symptoms of Lassa fever (LF) (5 items)**			
Item 14 Symptoms of LF: Miscarriage and fever	0.984		
Item 12 Symptoms of LF: Swollen face and neck, and deafness	0.983		
Item 10 Symptoms of LF: Proteinuria and nasal bleeding	0.97		
Item 11 Symptoms of LF: Elevated creatinine levels and abdominal pain	0.969		
Item 13 Symptoms of LF: Chest and muscle pains, and headache	0.907		
**Factor 2 (F2): Transmission of Lassa fever (7 items)**			
Item 7 Sources of LF transmission		0.915	
Item 3 Transmission of LF via person to person		0.903	
Item 5 Transmission of LF via sexual intercourse		0.897	
Item 2 Transmission of LF via a vector		0.842	
Item 4 Transmission of LF via asymptomatic patients		0.81	
Item 1 Causative agent of LF		0.779	
Item 6 Transmission of LF via patient’s corpse		0.726	
**Factor 3 (F3): Lassa fever prevention and control practices (6 items)**			
Item 16 Types of personal protective equipment (PPE)			0.768
Item 17 Best practices of wearing a PPE			0.742
Item 18 Best practices of performing a hand hygiene			0.638
Item 15 A statement about LF prevention and control practices			0.551
Item 8 Protection from LF via vaccine			-0.512
Item 9 Control and treatment of LF with ribavirin			-0.493

### Knowledge of Lassa fever and its prevention and control measures among the participants

Overall, mean knowledge score of LF and its PC measures was 11.60 (64.42%) (standard deviation ±8.14) and the range was from 3 to 18 (following a non-significant Kolmogorov-Smirnov value). Specifically, forty-eight percent (193/400) of the participants had adequate (good) knowledge of LF; while the rest (51.8%; 207/400) had poor (inadequate) knowledge. Table 4 showed the knowledge of participants on LF. The highest correct response scores were for items 17 (best practice in wearing a personal protective equipment (PPE)-0.92±0.28 (92.0%)), 16 (types of PPEs-0.84±0.36 (84.0%)), 15 (LF prevention and control measures-0.77±0.0.42 (77.0%), 1 (causative agent of LF-0.75±0.43 (75.0%)) and 2 (transmission of LF by its vector-0.75±0.43 (75.0%)). The least correct response scores were for items 18 (best practice in performing a hand hygiene-0.20±0.40 (20.0%)), 8 (vaccine for protection from LF 0.53±0.50 (53.0%)), 9 (effectiveness of ribavirin in the treatment and control of LF-0.53±0.50 (53.0%)), 6 (LF transmission via corpse of LF patient-0.57±0.50 (57.0%)) and 7 (sources of LF transmission -0.58±0.49 (58.0%)).

**Table 4:** Knowledge of Lassa fever symptoms, transmission and its prevention and control measures among healthcare workers during an outbreak in Katsina (N = 400).

Items	Correct options	Correct answers (n,%)	Incorrect answers (n,%)	Unsure answers (n, %)	Mean knowledge score (%)
1. LF is caused by:	c	299 (74.8)	31 (7.8)	70 (17.5)	0.75±0.43 (75.0%)
a. Bacteria b. Fungí c. Virus					
d. Protozoa e. Not sure					
2. LF is transmitted by:	c	299 (74.8)	18 (4.5)	83 (20.8)	0.75±0.43 (75.0%)
a. Flies b. Mosquitoes c. Rodents					
d. Not sure					
3. Can LF be transmitted from one person to another?	a	245 (61.3)	82 (20.5)	73 (18.3)	0.61±0.49 (61.0%)
a. Yes b. No c. Not sure					
4. LF CANNOT be transmitted when the patient is NOT having symptoms	b	282 (70.5)	84 (21.0)	34 (8.5)	0.71±0.46 (71.0%)
a. Yes b. No c. Not sure					
5. Can LF be transmitted through sexual intercourse?	a	235 (58.8)	82 (20.5)	83 (20.8)	0.59±0.49 (59.0%)
a. Yes b. No c. Not sure					
6. Is the corpse of an LF patient infectious?	a	229 (57.3)	136 (34.0)	35 (8.8)	0.57±0.50 (57.0%)
a. Yes b. No c. Not sure					
7. LF patients, their visitors, their healthcare workers, medical equipment and the hospital environment are the sources of LF transmission	a	232 (58.0)	40 (10.0)	128 (32.0)	0.58±0.49 (58.0%)
a. Yes b. No c. Not sure					
8. Vaccines can protect one from contracting LF.	a	210 (52.5)	37 (9.3)	153 (38.3)	0.53±0.50 (53.0%)
a. Yes b. No c. Not sure					
9. Ribavirin can be effective in the treatment and control of LF.	a	211 (52.8)	36 (9.0)	153 (38.3)	0.53±0.50 (53.0%)
a. Yes b. No c. Not sure					
**In which of the following situations would you suspect LF as been responsible for the symptoms the patient is experiencing:**					
10. A patient presented with fever, vomiting, nasal bleeding and proteinuria	a	268 (67.0)	23 (5.8)	109 (27.3)	0.67±0.47 (67.0%)
a. Yes b. No c. Not sure					
11. A patient presented with generalized body weakness, diarrhoea, abdominal pain and elevated creatinine levels	a	262 (65.5)	28 (7.0)	110 (27.5)	0.66±0.48 (66.0%)
a. Yes b. No c. Not sure					
12. A patient presented with swollen face and neck, sore throat, and hearing loss	a	263 (65.8)	24 (6.0)	113 (28.3)	0.66±0.48 (66.0%)
a. Yes b. No c. Not sure					
13. A patient presented with generalized body weakness, chest pain, headache and muscle pain	a	249 (62.3)	23 (5.8)	128 (32.0)	0.62±0.49 (62.0%)
a. Yes b. No c. Not sure					
14. A pregnant woman who had just returned from Edo State, had a miscarriage and fever	a	263 (65.8)	23 (5.8)	114 (28.5)	0.66±0.48 (66.0%)
a. Yes b. No c. Not sure					
15. Which of the following statements about IPC on LF is NOT true?	a	309 (77.3)	10 (2.5)	81 (20.3)	0.77±0.42 (77.0%)
a. IPC increases the prevalence of Lassa fever					
b. IPC reduces the number of Lassa fever-related deaths					
c. IPC leads to safer wards and healthcare facilities					
d. IPC prevents antimicrobial resistance					
e. Not sure					
16. Which of the following is NOT a PPE?	d	337 (84.3)	16 (4.0)	47 (11.8)	0.84±0.36 (84.0%)
a. Apron b. Boot c. Respirator					
d. Ventilator e. Not sure					
17. Which of the following is NOT a best practice of wearing of a PPE?	a	367 (91.8)	32 (8.0)	1 (0.3)	0.92±0.28 (92.0%)
a. Wearing a gown outside the environment of one’s duty post					
b. Performing hand hygiene before glove use					
c. Performing hand hygiene after glove use					
d. Wearing goggle for high-risk procedure on an LF patient					
e. Not sure					
18. Which of the following is NOT a best practice in performing a hand hygiene?	d	78 (19.5)	319 (79.8)	1 (0.3)	0.20±0.40 (20.0%)
a. Each time before touching an LF patient					
b. After contact with an LF patient					
c. After contact with blood and body fluid of an LF patient					
d. Use of hand sanitizer when hands have been visibly soiled					
e. Not sure					
**Overall average knowledge score**					**11.60±8.14 (64.4%)**

n, frequency; %, percentage; LF, Lassa fever; IPC, infection prevention and control

### Significant differences detected among subgroups

Chi-square tests for independence indicated the following significant associations between independent variables and the *first* dependent categorical variable (having adequate or inadequate LF knowledge): occupational categories of HCWs -χ^2^ (8, n = 400) = 46.11, *p* < 0.0005, Cramer’s V = 0.34; place of practice-χ^2^ (3, n = 400) = 11.82, *p* = 0.008, Cramer’s V = 0.17; educational status-χ^2^ (1, n = 400) = 5.23, *p* = 0.022, Cramer’s V = 0.11 and LF training status-χ^2^ (1, n = 400) = 17.62, *p* < 0.0005, Cramer’s V = 0.25.Chi-square tests for independence also indicated the following significant associations between independent variables and the *second* dependent categorical variable (levels of LF knowledge differentials-no overestimation and over-estimation): age group-χ^2^ (3, n = 276) = 20.08, *p* < 0.0005, Cramer’s V = 0.27; years of practice-χ^2^ (2, n = 276) = 9.80, *p* = 0.007, Cramer’s V = 0.19; and LF training status-χ^2^ (1, n = 276) = 19.46, *p* < 0.0005, *Phi* coefficient = 0.27.

The results for the LF knowledge differentials were: LF knowledge underestimators-86; Accurate LF knowledge estimators-100 and LF knowledge overestimators-90. However, to put these results into proper perspectives and validate it, HCWs designations/occupational categories were used to stratify the reclassified self-perceived LF knowledge scores (High and Low) and actual LF knowledge scores (Inadequate and Adequate) by cross-tabulation (Table 5).

**Table 5:** Differences in the levels of LF knowledge among healthcare workers who were aware of the LF outbreak (N = 276).

Perceived knowledge	Actual knowledge		
	Inadequate	Adequate	
	Frequency (%)	Frequency (%)	Total
Low	53 (37.1)	86 (64.7)	139 (50.4)
High	90 (62.9)	47 (35.3)	137 (49.6)
Total	143 (51.8)	133 (48.2)	276 (100)

The resultant Table showed three types of LF knowledge estimators: accurate estimators, under-estimators and over-estimators with all the nine different HCWs’ designations having members in each of the three types of LF knowledge estimations. Accurate LF knowledge estimators comprised 36.2% (100/276) of those who were trained on LF and were characterized by those who believed (predicted and in reality) they were below average (53%; 53/100) or above average (47%; 47/100). The top three HCWs in this category were: medical doctors-22%, 22/100; nurses-18%, 18/100 and midwives-16%, 16/100. The over-estimators constituted 32.6% (90/276) of those who were trained on LF and were those whose perceived themselves to be *very* knowledgeable on LF and its PC measures but in reality, were much *less* knowledgeable. Here, the top three categories of HCWs were: nurses-15; CHEWs-15 and midwives-13. Approximately one in three (31.2%; 86/276) LF- trained participants under-estimated their knowledge of LF and its PC measures. This group belonged to those who perceived their LF knowledge to inadequate but in reality, it was adequate. Pharmacists (22), nurses (21) and medical doctors (17) were the three dominant members in this category. Overall, sixty-four percent of (176/276) LF- trained HCWs indulged in the bias of flawed self-assessment of their LF knowledge, which is characterized by underestimation (31.2%; 86/276) and overestimation (32.6%; 90/276) of their LF knowledge with nurses being among the top two in either category.

### Predictors of knowledge of Lassa fever among the participants

Medical doctors were more than twice (2.40; 95% CI:1.10–5.22; *p* = 0.03) likely to be more knowledgeable about LF than other professionals (Table 6). Participants who practice in a tertiary care center were about twice (1.82; 95% CI:1.07–3.08; *p* = 0.03) likely to be more knowledgeable about LF than their counterparts in secondary care centers, while those who received formal training on LF were twice (2.06; 95% CI:1.19–3.57; *p* = 0.009) likely to be more knowledgeable about LF than those who did not. Table 7 showed that only LF training status of participants predicted LF knowledge overestimation. Those who received LF training were more than twice (2.53; 95% CI:1.49–4.30; *p* = 0.001) likely to indulge in the bias of LF knowledge overestimation than their counterparts who did not.

**Table 6:** Predictors of knowledge of Lassa fever and its prevention and control measures among healthcare workers during an outbreak in Katsina, Nigeria (N = 400).

Predictors	B	S.E.	Wald	df	P value^1^	Odds ratio	95% CI for odds ratio
LF training status	0.725	0.279	6.74	1	0.009	2.064	1.194–3.566
HCW’s cadre	0.875	0.397	4.858	1	0.028	2.398	1.102–5.220
Place of work	0.599	0.268	4.977	1	0.026	1.82	1.075–3.079
Educational status	0.244	0.274	0.796	1	0.372	1.277	0.746–2.184
Constant	-0.875	0.224	14.67	1	0	0.424	

^1^ Bold P values are for significant (p< 0.05) predictors; CI, Confidence interval

**Table 7:** Predictors of knowledge overestimation of Lassa fever and its prevention and control measures among healthcare workers during an outbreak in Katsina, Nigeria (N = 276).

Predictors	B	S.E.	Wald	df	P value^1^	Odds ratio	95% CI for odds ratio
LF training status	0.929	0.27	11.866	1	0.001	2.533	1.493–4.297
Age	0.024	0.032	0.559	1	0.454	1.024	0.962–1.091
Years of practice	0.036	0.037	0.91	1	0.34	1.036	0.963–1.115
Constant	-1.471	0.834	3.107	1	0.078	0.23	

1 Bold p value is for a significant (p<0.05) predictor; CI-Confidence interval

## Discussion

This survey is probably the first in Africa to assess LF knowledge overestimation among HCWs including evaluation of psychometric properties and underlying factor-structure of the survey instrument. It is also among the *very few* studies from Northern Nigeria that focused on LF knowledge level HCWs required to either manage LF patients or prevent LF outbreaks. Findings from the survey showed that participants answered three out of every five questions on LF and its PC measures correctly and this was significantly predicted by participants’ LF training status, place of work and occupational category. In addition, participants’ LF training status was the only participant characteristic that predicted LF knowledge overestimation. Although LF knowledge overestimation was observed among about one in four participants, each HCW occupational category had members that indulged in the LF knowledge overestimation. This is consistent with the trend reported by another study involving infectious disease [50]. Knowledge overestimation is an undesirable phenomenon that needs to be seriously considered and urgently addressed. This is because HCWs’ exaggeration of their knowledge of a disease increases the propensity to misdiagnose [50,51] or mismanage [49] and this can potentially compromise patient outcomes and safety. In addition, knowledge overestimation among individuals has been also linked with an increased likelihood of non-adherence to infectious diseases safety guidelines [48], and this is probably associated with such individuals’ unrealistic optimism about their own health risks compared with those of others [51]. The current study showed that nurses and CHEWs exhibited above-average effect (LF knowledge overestimation) the most, and this is similar to the finding reported by Ameade *et al* [49]. Therefore, there is a clear need to identify and deploy strategies that will modulate HCWs’ exaggeration of LF knowledge. For instance, the use of personalized feedback [51] which involves requesting HCWs to initially self-rate their knowledge of LF and its PC measures followed by a pre-test, LF training, post-tests with interactions between facilitators and the HCWs, followed by HCWs’ re-assessment of their LF knowledge. This strategy may make HCWs more cautious in their estimation and reduce exaggeration of LF knowledge. In addition, the use of bolstered self-esteem which involves initially requesting HCWs to self-rate their LF knowledge and then shown a video of the consequences of knowledge overestimation before being asked to reassess their knowledge may also promote self-accuracy in the rating of LF knowledge [51].

Our finding showing that the majority of HCWs rated their knowledge of LF as low (51.4%) is consistent with that of Alsahafi and Cheng who reported that the health workforce in Saudi Arabia rated their knowledge of MERS Coronavirus and other emerging infectious diseases as low [52]. Therefore, there is a clear need for regular audit of HCWs’ knowledge of LF so as to identify relevant gaps, and develop and implement appropriate countermeasures to close the identified knowledge gap.

Our study showed a higher proportion of HCWs with adequate LF knowledge compared to that reported by Chinaka five years ago (8% *vs* 64%) [24] and this difference may be attributable to the efforts of the NCDC in sensitizing the state’s health workforce on LF, and the organization of zonal and national workshops on LF case management as a part of outbreak response. In addition, the recent update of the LF management guidelines to prioritize standard precautions and LF prevention and control may also be contributory [22]. It is also possible that being the *second outbreak* in the state, the level of awareness and knowledge of LF among the HCWs might have increased through activities of the state’s department of public health in the state’s ministry of health.

Higher preponderance of frontline HCWs: medical doctors, nurses/midwives and laboratory scientists, and proportion of participants with previous exposure to LF training and LF-specific personal protective equipment training in a study have been linked with higher level of LF knowledge among HCWs [30]. These probably explained why other surveys that assessed LF knowledge among HCWs in Northern Nigeria, specifically Kaduna (doctors-5 *vs* 61; nurses-15 *vs* 81 and midwives-7 *vs* 56)) [32] and Plateau state (doctors-45 *vs* 61) [53], recorded lower LF knowledge levels than those in our study.

Similarly, the LF knowledge level of the participants in our study was also better than that reported among HCWs surveyed in Enugu [36], Edo [29], Ondo [30] and Ebonyi [54]. HCWs in privately-owned health facilities and primary health facilities tend to have less level of LF knowledge in Southern Nigeria [29]. This may explain why the present study recorded better level of LF knowledge as its participants were from government-owned secondary and tertiary health facilities. In addition, the present study was conducted during outbreaks of both LF and coronavirus disease 2019; thus, the participants’ level of awareness and information-seeking is likely to be higher than their counterparts surveyed when there were no outbreaks [29,30,36] or only LF outbreak [54].

However, some studies that assessed the LF knowledge among HCWs in Ondo state [28,47] reported relatively higher level of LF knowledge. This is not surprising as Ondo state has the second highest LF burden in Nigeria and experiences numerous LF outbreaks [47]. Consequently, participants in the reference studies are likely to be more familiar with LF-related matters than those surveyed by the present study who experienced LF outbreak only for the second time. This might have contributed to the higher level of LF knowledge recorded among the HCWs surveyed in the reference studies.

Further, the present study found the HCWs surveyed to have poor knowledge of hand hygiene. Hands have been recognized as the main pathways of pathogen transmission while a patient is receiving healthcare [55]. Thus, LF training and LF-specific personal protective equipment trainings need to prioritize hand hygiene in the design of appropriate intervention focused on improving HCWs’ knowledge of LF prevention and control measures.

### Limitation

Our study was a multi-centre, cross-sectional assessment, had the largest sample size among LF surveys (with similar objectives) conducted in the country and included often under-represented HCWs such as the pharmacists, dentists and radiographers. However, it may be limited by some inherent biases associated with surveys. This might include social desirability bias which could result in overrepresentation of the responses of respondents who are more likely to be knowledgeable about LF and its PC measures. However, the mean LF knowledge score obtained showed that the study is likely to be free of this bias, as otherwise the knowledge score would have been higher than that observed in the present study. In addition, majority of the respondents rated their LF knowledge as poor, and this seems inconsistent with the behavior of those who have social desirability traits. Recall bias might also have been introduced when respondents were requested to provide information on past training received on LF and its PC measures or global rating of LF knowledge. Data collection during an LF outbreak ensures matters related to LF are at the forefront and topical, thus may not be difficult to recall. Purposive sampling technique may introduce selection bias into a study. However, we minimized this bias by the use of multiple study sites, *a priori* determination of sample size; and inclusion of all HCWs that are relatively few in numbers (dentists, medical laboratory scientists and radiographers) and stratified random sampling for the other categories of HCWs. Hence, we are confident that we obtained a group of participants that is probably representative and this may have improved the generalizability of the study findings.

### Conclusion

The level of knowledge of LF and its PC measures among the HCWs surveyed was suboptimal and participants’ LF training status, place of work and occupational category were the most significant predictors of the knowledge level. In addition, LF knowledge overestimation was observed among each category of HCWs surveyed and this was also significantly predicted by LF training status.

These knowledge and training gaps might have significant negative public health implications especially in mitigating the nosocomial spread of LF, prevention of infection with LASV among HCWs and reduction of avoidable clinical and financial burden due to LASV in Nigeria. Further, having knowledgeable human resources for health may also help in the control of other epidemic-prone zoonotic viral diseases that are presently ravaging at least half of the states in Nigeria.

## Supporting information

S1 Questionnaire Knowledge of Lassa fever, its prevention and control practices and their predictors among healthcare workers in Northern Nigeria. (DOCX)
