# Supplementary material for: Knowledge of Lassa fever, its prevention and control practices and their predictors among healthcare workers during an outbreak in Northern Nigeria: A multi-centre cross-sectional assessment
Source: PLoS Negl Trop Dis. 2022 Mar 14;16(3):e0010259. doi: 10.1371/journal.pntd.0010259 (PMC9045733; doi:10.1371/journal.pntd.0010259)
Supplement: S1 Questionnaire — (DOCX) [file pntd.0010259.s001.docx]

**Knowledge of Lassa fever, its prevention and control practices and their predictors among healthcare workers in Northern Nigeria**

**HREC reference number:** MOH/ADM/SUB/1152/1/341

**Contact:** Yusuf Hassan Wada; [hasawa2011@gmail.com](mailto:hasawa2011@gmail.com); 08069688246

**CONSENT FORM**

You are invited to take part in a study entitled **Knowledge of Lassa fever, its prevention and control practices and their predictors among healthcare workers in Northern Nigeria.** Your participation in the study is voluntary.

Please note that all the information provided will be treated with utmost confidentiality and findings will be used solely for research purposes.

 Please **tick (√)** if you agree to take part in this study: YES NO

**NB:** LF represents Lassa Fever

**QUESTIONNAIRE**

**SECTION A: SOCIODEMOGRAPHIC DATA**

1. Age (years)………………………..
2. Gender: Male Female
3. Profession: Medical Doctor Pharmacist Nurse Medical Lab. Radiographer

CHEWS Dentist Others (please specify)

1. Year(s) of practice……………………………… 5. Respondent’s name of institution:…………………
2. Marital Status: Single Married Divorced/Widowed
3. Educational Status: Diploma and Below Degree and Above

**SECTION B: KNOWLEDGE & AWARENESS OF RESPONDENTS ABOUT LASSA FEVER**

(Please tick (√) only one option)

1. Are you aware of ongoing Lassa Fever (LF) outbreak in Nigeria? Yes

No

1. Have you ever attended a training program or presentation on LF and its prevention and control measures?

Yes No

1. If yes to Q9 above (if no to Q9 above, move to Q11), how would you rate your knowledge of Lassa fever?

1 (poor) 2 (average) 3 (good) 4 (very good) 5 (excellent)

1. LF is caused by: Bacteria Fungi Virus Protozoa I don’t know
2. LF is transmitted by: Flies Mosquitoes Rodents I don’t know
3. Can LF be transmitted from one person to another?

Yes No I don’t know

14. LF CANNOT be transmitted from the patient who is NOT having symptoms. Yes No I don’t know

1. Can LF be transmitted through sexual intercourse?

Yes No I don’t know

1. Is the corpse of an LF patient infectious?

Yes No I don’t know

1. LF patients, their visitors, their healthcare workers, medical equipment and the hospital environment are the sources of LF transmission.

Yes No I don’t know

18. Can a vaccine protect one from contracting LF?

Yes No I don’t know

1. Can ribavirin be effective in the treatment and control of LF?

Yes No I don’t know

***Which of the following scenarios would you suspect LF as being likely responsible for what the patient is experiencing*** (Please tick (√) only one option)***:***

1. A patient presented with fever, vomiting, nasal bleeding and proteinuria

Yes No I don’t know

1. A patient presented with generalized body weakness, diarrhoea, abdominal pain and elevated creatinine levels

Yes No I am not sure

1. A patient presented with swollen face and neck, sore throat, and hearing loss/deafness.

Yes No I am not sure

1. A patient presented with generalized body weakness, chest pain, headache and muscle pain.

Yes No I am not sure

1. A pregnant woman who had just returned from Edo State, had a miscarriage and fever

Yes No I am not sure

1. Which of the following statements about IPC on LF is NOT true? (Please tick (√) only one option)

IPC increases the prevalence of lassa fever

IPC reduces the number of lassa fever-related deaths

IPC leads to safer wards and healthcare facilities

IPC prevents antimicrobial resistance

1. Which of the following is NOT a personal protective equipment (PPE) (Please tick (√) only one option)?

Apron Boot Respirator Ventilator

1. Which of the following is NOT a best practice of wearing of a PPE? (Please tick (√) only one option)

Wearing a gown outside the environment of one’s duty post

Performing hand hygiene before glove use

Performing hand hygiene after glove use

Wearing goggle for high-risk procedure on an LF patient

1. Which of the following is NOT a best practice in performing a hand hygiene? (select only ONE option)

Each time before touching an LF patient

After contact with an LF patient

After contact with blood and body fluid of an LF patient

Use of hand sanitizer when hands have been visibly soiled
